# Supplementary figures and images for: Fast Spiking Interneurons Autonomously Generate Fast Gamma Oscillations in the Medial Entorhinal Cortex with Excitation Strength Tuning ING–PING Transitions
Source: eNeuro. 2026 Jan 30;13(2):ENEURO.0452-25.2026. doi: 10.1523/ENEURO.0452-25.2026 (PMC12884686; doi:10.1523/ENEURO.0452-25.2026)

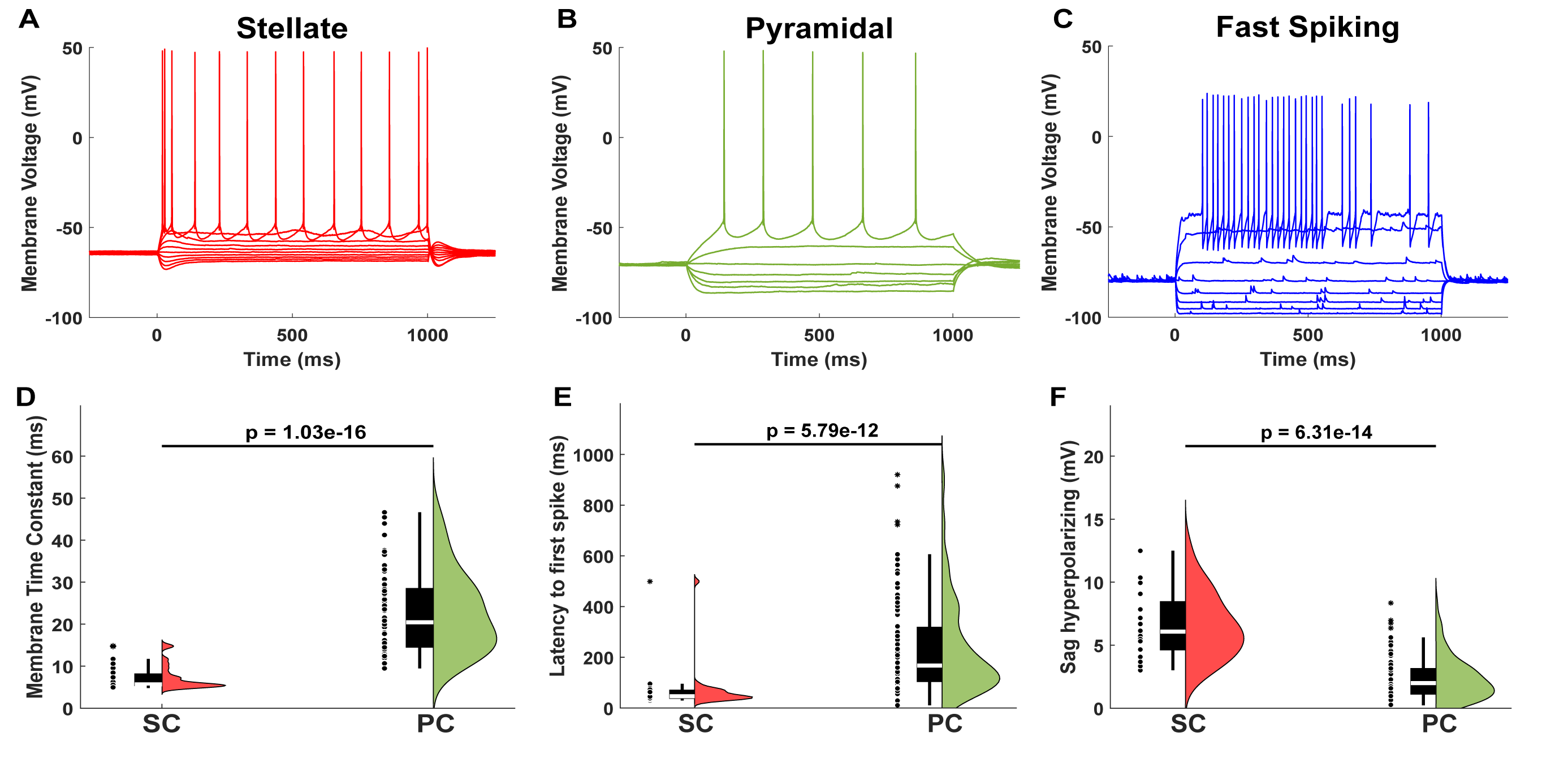

Supplement: Figure 1-1 — Classification of major electrophysiological cell types in mEC. A) Example voltage response to current steps in stellate cell. B) Example voltage response to current steps in pyramidal cell. C) Example voltage response to current steps in fast-spiking interneuron. D) Membrane time constants of stellate and pyramidal cells. Stellate cells have shorter time constants. E) Latency to first spike of stellate and pyramidal cells. Stellate cells fire sooner than pyramidal cells. F) Hyperpolarizing sag potential in stellate and pyramidal cells. Stellate cells have larger hyperpolarizing sag potentials. Download Figure 1-1, TIF file. [file eneuro-13-ENEURO.0452-25.2026-s001.tif]

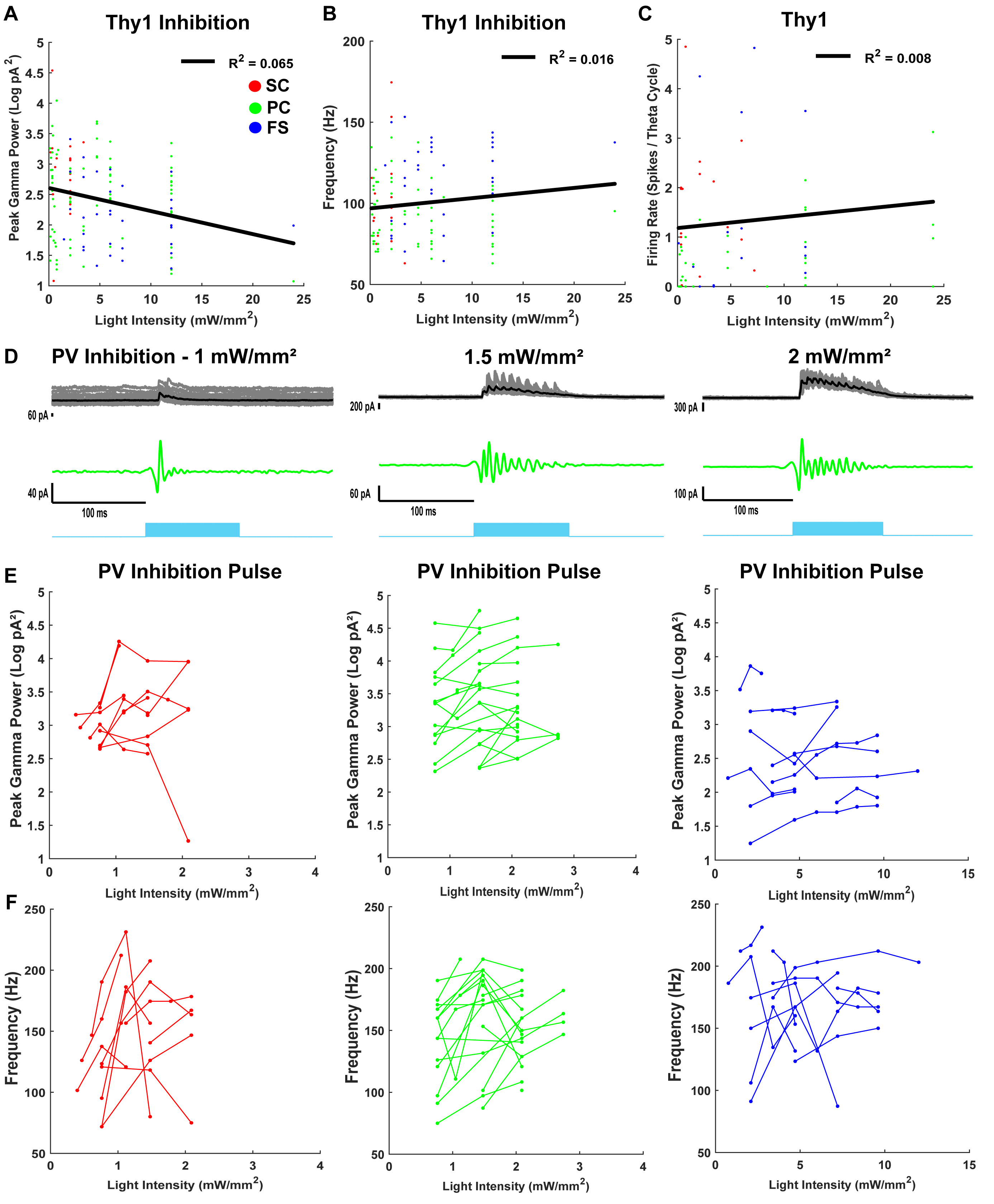

Supplement: Figure 3-1 — The effects of optogenetic stimulation intensity on mEC gamma activity. A) Peak log gamma power of inhibitory currents recorded from stellate cells, pyramidal cells, and fast-spiking interneurons in the mEC vs. peak light intensity of sinusoidal optogenetic Thy1 stimulation. B) Peak gamma frequency of inhibitory currents recorded from stellate cells, pyramidal cells, and fast-spiking interneurons in the mEC vs. peak light intensity of sinusoidal optogenetic Thy1 stimulation. C) Firing rates of stellate cells, pyramidal cells, and fast-spiking interneurons in the mEC vs. peak light intensity of sinusoidal optogenetic Thy1 stimulation. D) Example inhibitory current recordings in pyramidal cell during different levels of pulsed optogenetic PV stimulation (left: 1 mW/mm2, middle: 1.5 mW/mm2, right: 2 mW/mm2). Black line shows average inhibitory currents (20 trials). Gray lines show individual trials. Top traces are raw data. Middle traces are filtered from 50-250 Hz. Light blue square indicates stimulation period. E) Peak gamma power in paired individual cell recordings during different pulse PV stimulation intensities (left: stellate, middle: pyramidal, right: fast-spiking interneuron). Lines indicate paired cell recordings. F) Peak gamma frequency in paired individual cell recordings during different pulse PV stimulation intensities (left: stellate, middle: pyramidal, right: fast-spiking interneuron). Lines indicate paired cell recordings. Download Figure 3-1, TIF file. [file eneuro-13-ENEURO.0452-25.2026-s002.tif]

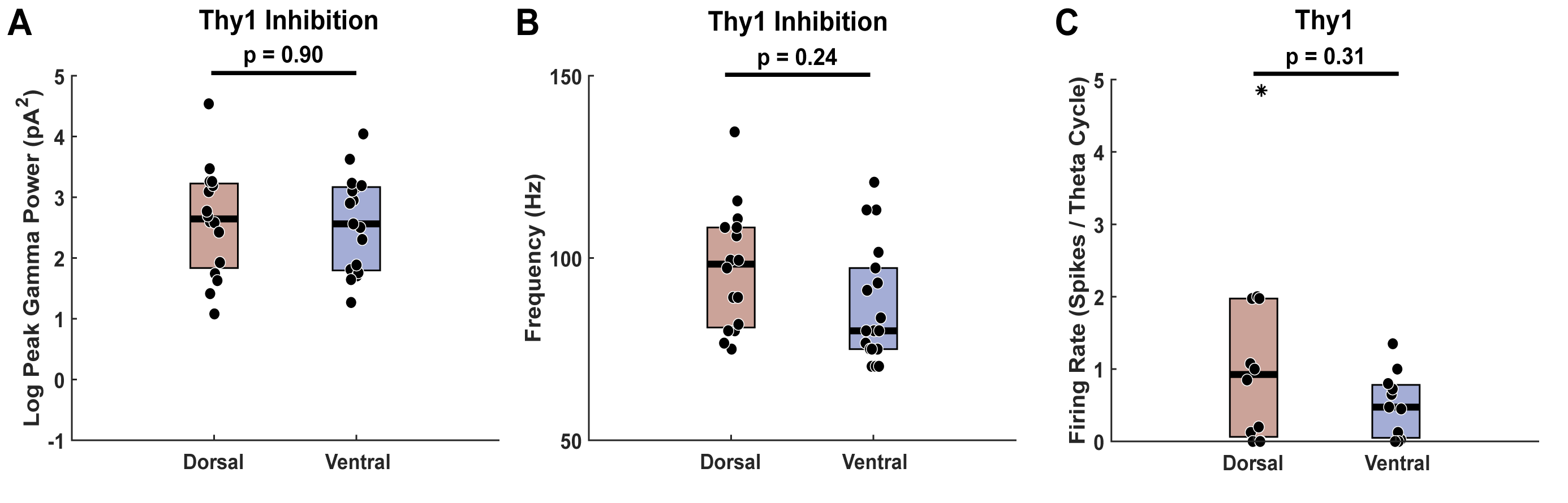

Supplement: Figure 3-2 — Gamma frequency inhibition is not different across the dorsal and ventral extents of the mEC during Thy1 stimulation. A) Peak log gamma power of inhibitory currents recorded from excitatory cells in the most dorsal and ventral mEC slices. B) Peak gamma frequency of inhibitory currents recorded from excitatory cells in the most dorsal and ventral mEC slices. C) Firing rates of excitatory cells in the most dorsal and ventral mEC slices. Download Figure 3-2, TIF file. [file eneuro-13-ENEURO.0452-25.2026-s003.tif]

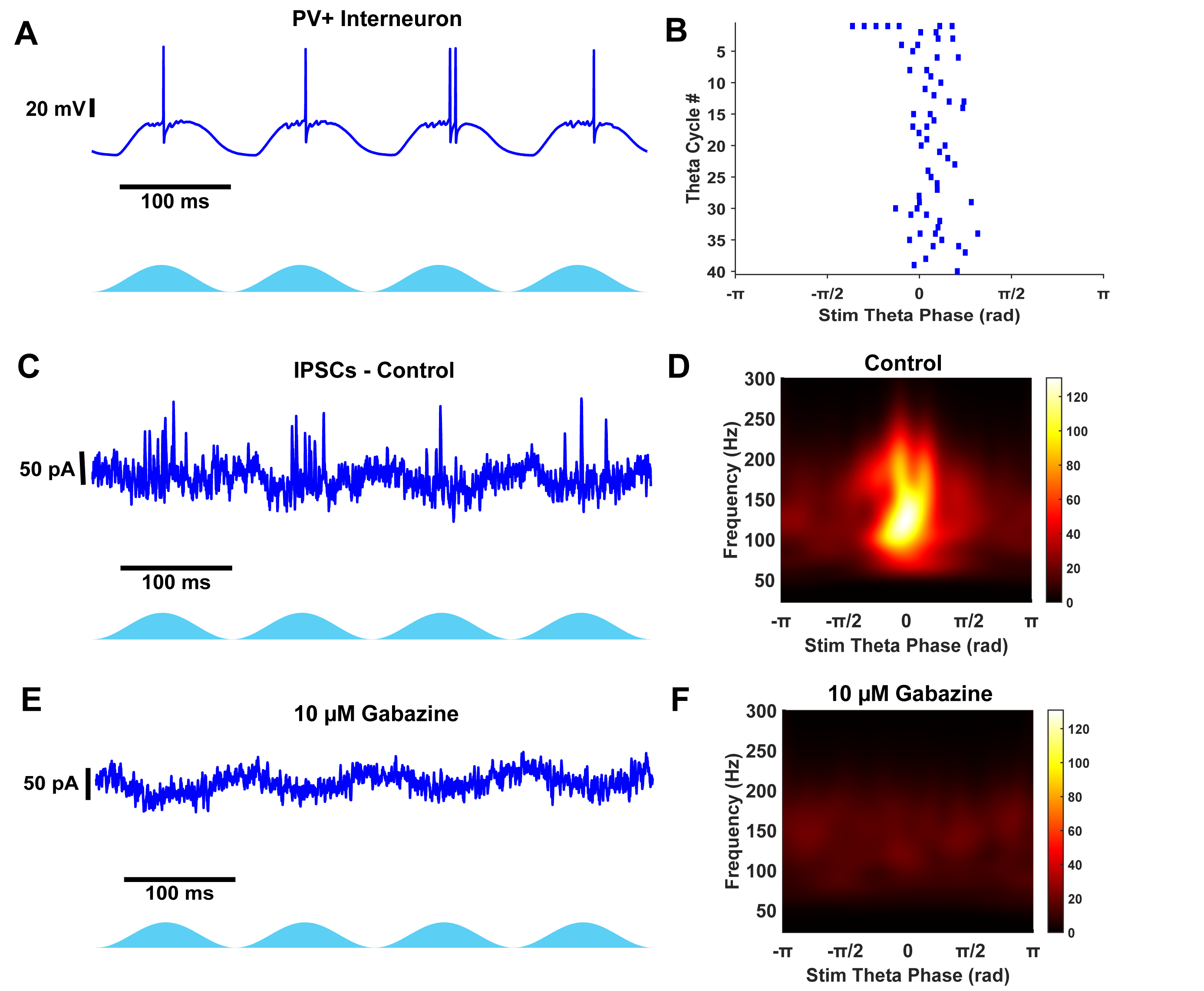

Supplement: Figure 5-1 — PV + interneuron receives fast GABAergic theta-nested gamma inhibition. A) Example voltage recording in PV + interneuron during network PV + optogenetic stimulation. B) Raster plot from 40 theta stimulation periods in same cell as A. C) Voltage clamp at 0 mV records theta-nested IPSCs during PV + stimulation. D) Average Scalogram from 40 theta stimulation periods of data from example in C. E) Voltage clamp at 0 mV observes no IPSCs after blocking GABAA receptors with 10 µM Gabazine. This data verifies theta-nested gamma in C is fast GABAergic inhibition. F) Average scalogram from 40 theta stimulation periods after blocking GABAA channels. Gamma frequency activity is abolished. Download Figure 5-1, TIF file. [file eneuro-13-ENEURO.0452-25.2026-s004.tif]

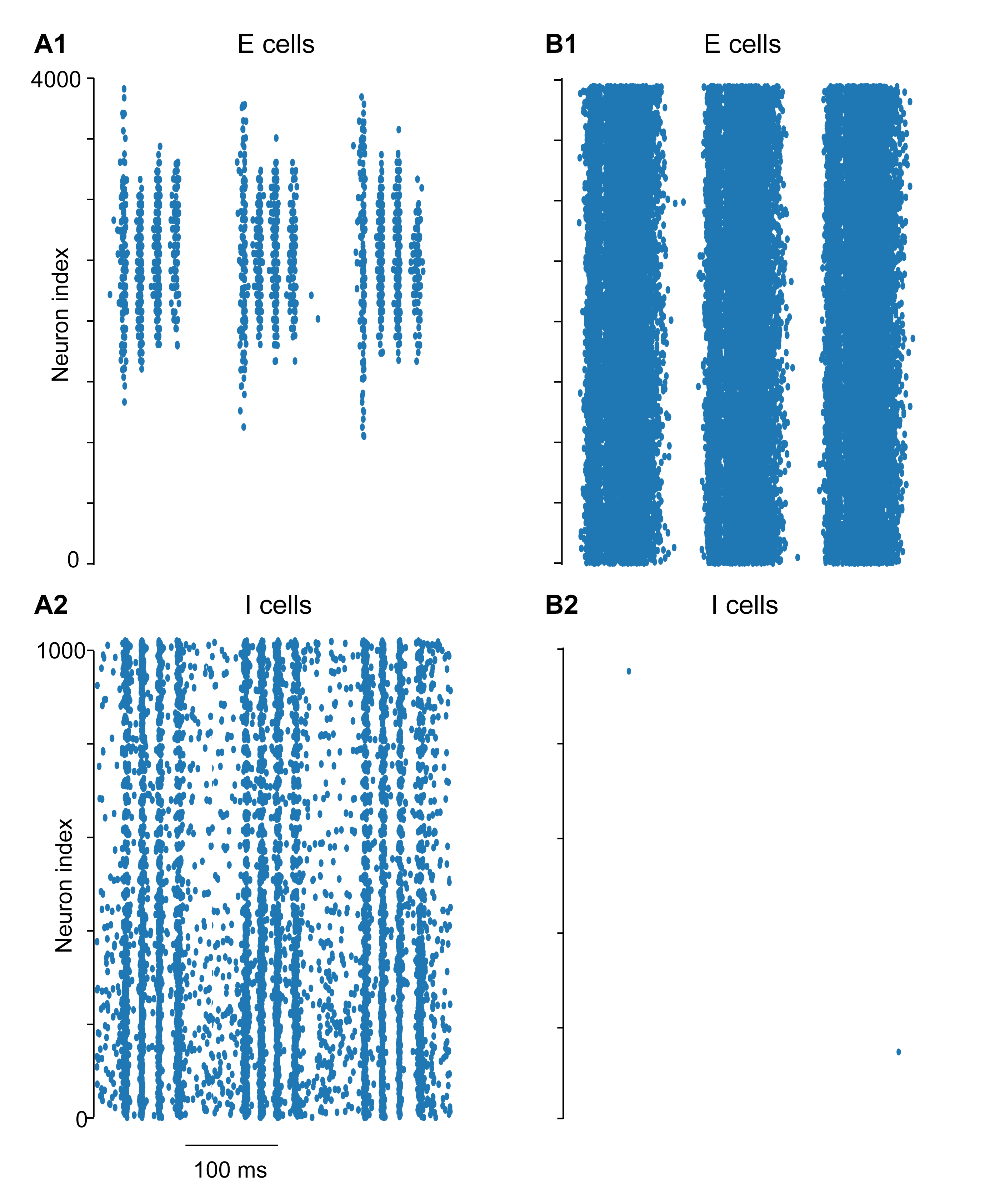

Supplement: Figure 6-1 — Simulations of theta-nested gamma oscillations using the grid cell model from Pastoll et al. (2013) with and without AMPA-mediated connections. A) Raster plots for (1) excitatory cells and (2) inhibitory cells with the default model parameters. B) Raster plots for (1) excitatory cells and (2) inhibitory cells with no AMPA-mediated connectivity. Original model: https://modeldb.science/150031?tab=2&file = GridCellModel/grid_cell_model and a revised simulation_fig_model.py file available at https://github.com/ccanav/pastoll_et_al_2013. Download Figure 6-1, TIF file. [file eneuro-13-ENEURO.0452-25.2026-s005.tif]

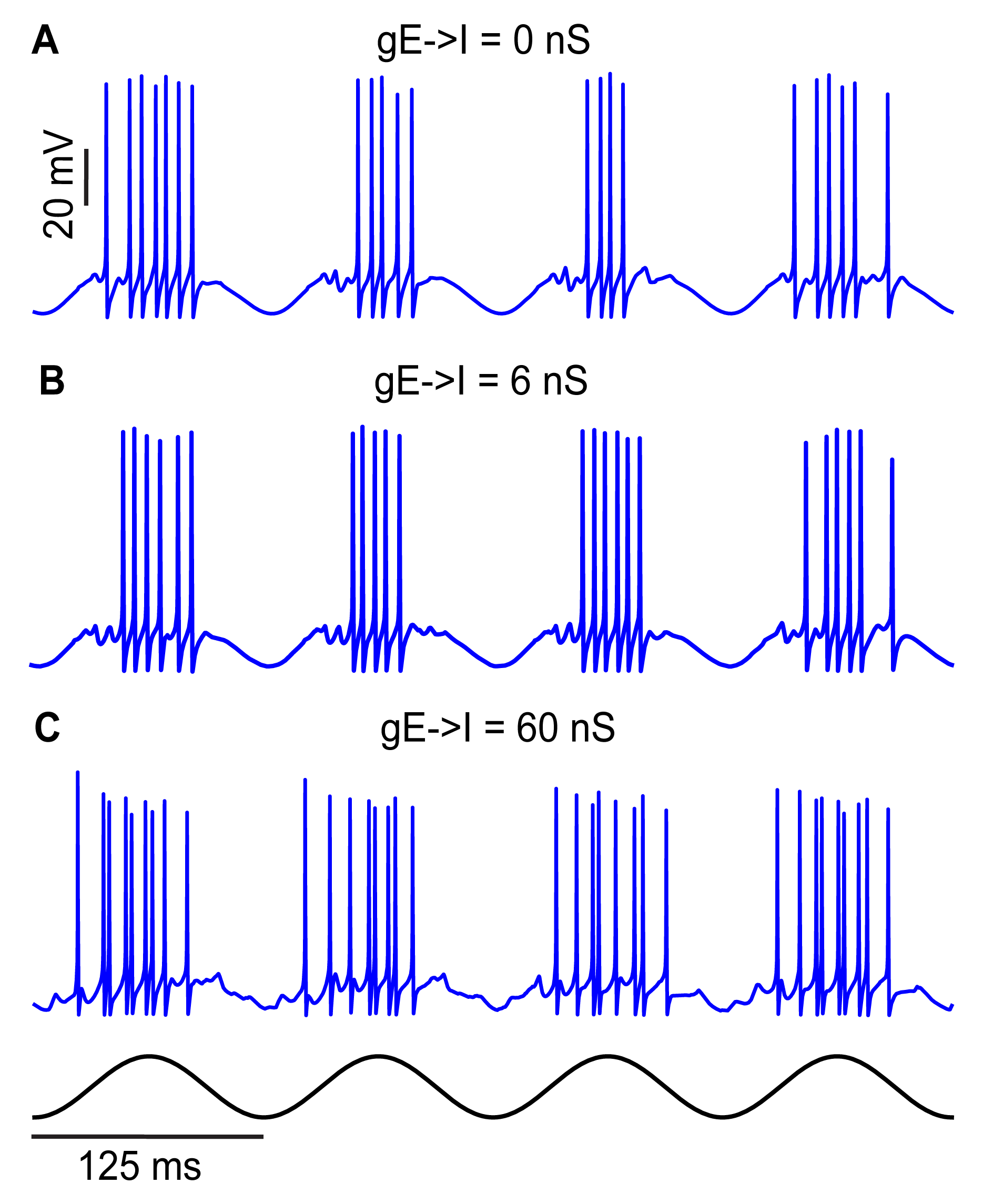

Supplement: Figure 6-2 — Simulated firing responses of PV interneurons for different E to I connection strengths. A, B) ING regime: With no synaptic excitation (top) and at weak excitation (middle), the I cells fire in a similar pattern. C) PING regime: With strong E to I conductance strength, PV interneuron firing is grouped into bursts, thereby increasing the latency to the next burst of excitation, and slowing the network gamma frequency. Download Figure 6-2, TIF file. [file eneuro-13-ENEURO.0452-25.2026-s006.tif]
